# Outcomes of Patients With Negative Cervical Imaging but Persistent Neck Tenderness Discharged With a Rigid Collar After Trauma

**DOI:** 10.7759/cureus.24170

**Published:** 2022-04-15

**Authors:** Melanie M Randall, John Egbert, Breanna M Ito, Jared E Yalung, Lance Brown

**Affiliations:** 1 Emergency Department, Loma Linda University Medical Center, Loma Linda, USA; 2 Pediatric Emergency Medicine Department, Loma Linda University Medical Center, Loma Linda, USA

**Keywords:** neck pain, cervical injury, patient experience, traumatic neck pain, cervical collar

## Abstract

Introduction

It is not uncommon for patients with persistent neck pain after trauma despite negative cervical imaging to be discharged with a rigid collar. Protocols for these patients vary widely. Few studies have evaluated clinical outcomes after discharge. No studies have evaluated the patient’s experience in a cervical collar after discharge.

Methods

We evaluated adults with blunt trauma and negative cervical spine imaging who were discharged in a rigid cervical collar. Over a 19-month period, 45 patients were available for analyses. The primary outcome was any identified missed injuries after discharge. Secondary outcomes were the incidence of patients self-clearing from their collars and complications related to wearing a collar.

Results

There were no missed traumatic injuries on follow-up imaging. Twenty of 45 patients cleared themselves from the collar without a physician order. Twenty-four patients had their collars removed by a provider in the clinic between 1-84 days after injury. One patient removed the collar after being advised by a chiropractor. More than half of patients reported one or more complications from wearing the cervical collar including pain, skin irritation, problems sleeping, difficulty talking or swallowing.

Conclusions

Collar complications are frequent. Follow-up imaging did not change outpatient management. Our data suggests against the practice of discharging trauma patients home in a cervical collar with negative imaging and no focal neurologic deficit.

## Introduction

It is not uncommon for patients with persistent traumatic neck pain despite negative initial imaging to be discharged home with a rigid cervical collar. The indications for this practice are unclear and can vary widely. Multiple validated decision rules exist to determine which patients require cervical imaging [1, 2]; however, these do not specify what treatment or follow-up should be performed in patients after negative imaging. Theologis et al. evaluated cervical spine clearance protocols in level one trauma centers and found that only half of the responding centers have established protocols [3]. For patients with persistent pain after negative imaging, 30% of these protocols recommend flexion-extension films. The rest include options such as magnetic resonance imaging (MRI), clinic follow-up, specialty consultation, collar maintenance, and collar discontinuation. Pekmezci et al. found similar variability in cervical spine clearance protocols for persistent neck pain among levels one, two, and three trauma centers in California [4].

There is morbidity associated with maintaining a cervical collar, with multiple studies demonstrating early and late side effects. In a study of healthy participants, all patients developed cervical pain within 30 minutes of immobilization [5]. Early on, collars can cause indentation marks and pressure ulcers [6-8]. Collars worn for greater than 24 hours have been independently associated with acquired pneumonia in patients older than 64 years [9]. There is also the question of whether the cervical collar can adequately protect the cervical spine from further injury. Its efficacy has not yet been evaluated in vivo [10].

Few studies have evaluated the outcomes of patients discharged with a cervical collar, and most have focused on outpatient MRI results. Dorney et al. reviewed pediatric patients with persistent tenderness and found that 2.1% had MRI findings related to injury on follow-up, but none required surgery [11]. Kongsted et al. prospectively followed 173 adults with neck pain and negative MRI imaging and showed that 0.5% of patients had traumatic cervical disc bulge or protrusion on repeat MRI imaging, but again, none required operative intervention [12]. The purpose of this study was to identify the outcomes of patients discharged home in a rigid cervical collar after negative imaging. The primary outcome was any identified missed injuries after discharge. Secondary outcomes included incidence of patients self-clearing from their collars, and self-reported complications related to wearing a collar.

## Materials and methods

Study setting and time period

We conducted a retrospective study of patients greater or equal to 18 years of age who presented to a level one trauma center and met the criteria for trauma team activation and evaluation. The study period was June 1, 2018 - December 31, 2019. This study was approved by the Loma Linda University Medical Center Institutional Review Board, approval number 5190114.

Study population

Patients with blunt trauma and negative cervical spine imaging results with persistent neck pain and who were discharged in a rigid cervical collar were included in the study. Negative cervical spine imaging results were defined as computed tomography (CT) reviewed by an attending radiologist with a final report of no fracture, dislocation, or major ligament tear. Patients with neurologic deficits, a Glasgow Coma Scale (GCS) score less than 14, or positive acute injury on imaging results were excluded.

Outcome measures

The primary outcome was any identified missed injuries after discharge. Secondary outcomes were incidence of patients self-clearing from their collars and self-reported complications related to wearing a collar.

Patient selection and follow-up

Patients were selected by querying our institution’s trauma database for patients that were discharged with a rigid cervical collar. We reviewed the medical records of all patients and obtained demographic, historical, and radiologic study information. We devised a standard questionnaire, which the research team developed after reviewing the literature and identifying data that could not be answered from the chart review. Trained volunteers made telephone calls to the patients to obtain follow-up information and data. After three unanswered calls on separate days, the subject was considered lost to follow-up. Individuals with Spanish listed as their primary language were contacted by a Spanish-speaking medical interpreter.

## Results

**Figure 1:**
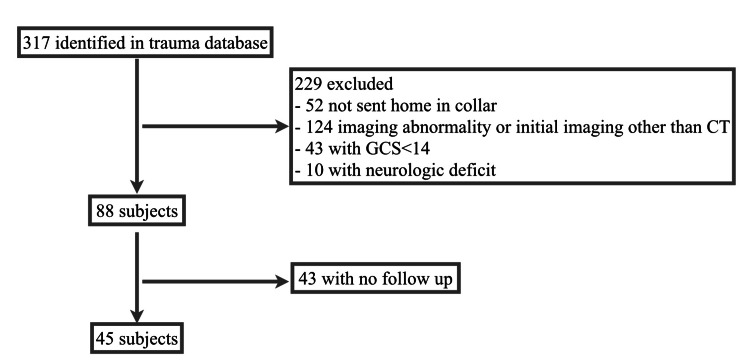
Figure subject identification GCS: Glasgow Coma Scale

A total of 317 patients were identified from our trauma database during the study period. Of these, 272 patients were excluded for the following reasons: 52 patients were not sent home in a collar, 113 patients had abnormal initial imaging, 43 patients had a GCS <14, 10 patients had neurologic deficits, 11 patients received initial imaging other than CT, and 43 patients were lost to follow-up or unable to be contacted (Figure 1). The remaining 45 patients were included in our analyses.

The median age was 60 years (IQR 41-69). Seventeen patients were male, and 28 patients were female. The mechanisms of injury included 25 patients in a motor vehicle crash, 17 patients with a fall, and three patients with a direct blow. All patients had CT of the cervical spine as the initial imaging modality. All CT results were negative for acute injury. There were 26 patients who were admitted to the acute care surgery (ACS) service, and 19 patients were discharged from the emergency department after trauma evaluation. Of the 26 patients admitted, 10 had an MRI of the cervical spine for persistent cervical pain. Two patients were found to have significant chronic or congenital cervical abnormalities unrelated to trauma and were referred to the spine clinic for future surgical evaluation.

Forty-three of 45 patients were referred for outpatient follow-up with ACS or spine clinic. The range of scheduled follow-up visits was one to six weeks. Fourteen patients did not follow up, and two patients did not have information available. Nine patients had outpatient follow-up imaging (Table 1). None of these patients had significant imaging findings that led to further intervention for traumatic injury.

**Table 1:** Patients with known outpatient cervical imaging y/o = years old; M = male; F = female; w/ = with; mm = millimeter; Fx = fracture; CT = computed tomography; DDD = degenerative disc disease; MRI = magnetic resonance imaging; MVC = motor vehicle crash; Flex/Ext = flexion / extension; AP/Lat = anterior posterior / lateral.

Age/ gender	Mechanism of injury	Cervical inpatient imaging	Cervical outpatient imaging	Cervical collar
48 y/o M	Direct blow	CT: Mild multilevel DDD, mild disc bulge C4-5	MRI negative	Physician removed after 7 days
32 y/o F	Direct Blow Jumped out of car	CT: reversal normal lordosis, paraspinal muscle spasm	MRI: degenerative changes from likely prior trauma, no acute injury	Physician removed after 42 days
54 y/o F	MVC	CT: possible chronic C6 spinous process fx MRI: normal	Flex/ex: normal	Physician removed after 13 days
29 y/o M	MVC	CT: normal	MRI “ligament tear” per patient, no report available	Chiropractor removed after 14 days
86 y/o M	Fall	CT: severe multilevel DDD w/central spinal stenosis at C3-4, C5-6, neuroforaminal narrowing	Flex/ex: 2 mm grade 1 anterolisthesis, possibly related to ligamentous laxity or injury. No acute displaced fracture. Moderate multilevel DDD. Neurosurgery diagnosed ligament laxity	Self-cleared after 7 days, no reported complications
18 y/o M	MVC	CT: normal	Flex/ex: mild neural foraminal narrowing C3-4	Physician removed after 14 days
60 y/o F	MVC	CT: mild DDD w/spinal canal stenosis at C2-3, C6-7; mild neuroforaminal narrowing	MRI: spinal canal narrowing, neuroforaminal narrowing, muscle strain	Physician removed after 14 days
63 y/o M	MVC	CT: multilevel DDD w/neuroforaminal narrowing	AP/Lat X-rays: neuroforaminal narrowing, no fracture	Physician removed after 14 days
41 y/o F	Direct blow Boxes fell on her	CT: multilevel mild posterior disc protrusions MRI: normal	Patient had unknown negative study, no report available	Self-cleared after 7 days, had a rash

Twenty-four patients had their collars removed by a physician in the clinic. For these patients, the time for wearing a collar ranged from one to 84 days (median 24 days, IQR 11-39.5). Twenty patients self-cleared from their collar without a physician's order. For this group, the time wearing a collar ranged from two to 40 days (median 14 days, IQR 7-21). One patient’s chiropractor recommended collar removal after 14 days.

More than half of the patients reported one or more complications from wearing the cervical collar. Twenty-two percent of patients reported collar pain, 20% of patients reported rash or skin irritation, 18% of patients reported problems sleeping due to the collar, and 16% of patients reported difficulty talking or swallowing.

## Discussion

This is the first study to focus on the patient experience of outpatient cervical collar compliance and complications after discharge. In our study, almost half of the patients removed their own cervical collars without consulting a physician. No patients in the study had significant traumatic findings on follow-up visits or imaging, and no patients underwent spinal surgery for missed or progressive traumatic injury. The incidence of reported collar-related complications was high.

Patients with persistent cervical pain incur a significant increase in healthcare costs including follow-up visits and further imaging [13]. In this study, only nine patients had follow-up outpatient imaging, none of which revealed any significant missed traumatic injury. There are numerous studies evaluating the utility of cervical MRI after a negative CT scan. These studies have wide variations in the incidence of significant MRI findings, from 0.5% to 44%; however, the incidence of MRI findings that require intervention is much smaller at 0% to 6% [12, 14-18]. While Malhotra et al. reported a high incidence of unstable findings on MRI, this was significantly more common in obtunded patients who were included in their study [18]. The most common findings on MRI were degenerative disc disease and spinal canal stenosis [14, 19]. The most common reason for surgical intervention in these patients is for advanced cervical spondylosis unrelated to the recent trauma.

Some studies have advocated for clearance of the cervical spine without MRI in awake and alert patients with persistent midline neck pain. This is proposed given the very low number of clinically significant findings on MRI if the CT is negative [15, 17, 19-21]. The Eastern Association for the Surgery of Trauma (EAST) guidelines state that there are several treatment options, but that data is limited [22]. Per the EAST guidelines, the cervical collar can be continued or removed after a negative MRI or removed after negative and adequate flexion-extension films.

Limitations of this study include its retrospective nature and loss of patients to follow-up. Further studies should consider prospective evaluation of the utility of maintaining a cervical collar at hospital discharge despite negative imaging. Our study suggests this practice is unnecessary.

## Conclusions

Our data suggests against the practice of discharging trauma patients home in a cervical collar with negative imaging and no focal neurologic deficit. Patients frequently experience collar side effects and often self-clear from their collars without a physician's order. Cervical imaging after discharge in patients with persistent neck pain and no significant initial traumatic findings does not appear to change management.

## References

[1] Hoffman JR, Mower WR, Wolfson AB, Todd KH, Zucker MI (2000). Validity of a set of clinical criteria to rule out injury to the cervical spine in patients with blunt trauma. National Emergency X-Radiography Utilization Study Group. N Engl J Med.

[2] Stiell IG, Wells GA, Vandemheen KL (2001). The Canadian C-spine rule for radiography in alert and stable trauma patients. JAMA.

[3] Theologis AA, Dionisio R, Mackersie R, McClellan RT, Pekmezci M (2014). Cervical spine clearance protocols in level 1 trauma centers in the United States. Spine (Phila Pa 1976).

[4] Pekmezci M, Theologis AA, Dionisio R, Mackersie R, McClellan RT (2015). Cervical spine clearance protocols in Level I, II, and III trauma centers in California. Spine J.

[5] Chan D, Goldberg R, Tascone A, Harmon S, Chan L (1994). The effect of spinal immobilization on healthy volunteers. Ann Emerg Med.

[6] Ham WH, Schoonhoven L, Schuurmans MJ, Leenen LP (2016). Pressure ulcers, indentation marks and pain from cervical spine immobilization with extrication collars and headblocks: an observational study. Injury.

[7] Liew SC, Hill DA (1994). Complication of hard cervical collars in multi-trauma patients. Aust N Z J Surg.

[8] Alvarez EM, del Ara Murillo Pérez M, Salobral Villegas MT, Caballero MD, Solanas MC, Fuentes CG (2004). Pressure sores secondary to immobilization with cervical collar: a complication of acute cervical injury (Article in Spanish). Enferm Intensiva.

[9] Nakanishi T, Mitra B, Ackland H, O'Reilly G, Cameron P (2019). Time in collars and collar-related complications in older patients. World Neurosurg.

[10] Deasy C, Cameron P (2011). Routine application of cervical collars--what is the evidence?. Injury.

[11] Dorney K, Kimia A, Hannon M (2015). Outcomes of pediatric patients with persistent midline cervical spine tenderness and negative imaging result after trauma. J Trauma Acute Care Surg.

[12] Kongsted A, Sorensen JS, Andersen H, Keseler B, Jensen TS, Bendix T (2008). Are early MRI findings correlated with long-lasting symptoms following whiplash injury? A prospective trial with 1-year follow-up. Eur Spine J.

[13] Ackland HM, Wolfe R, Cameron PA (2012). Health resource utilisation costs in acute patients with persistent midline cervical tenderness following road trauma. Injury.

[14] Ackland HM, Cameron PA, Varma DK (2011). Cervical spine magnetic resonance imaging in alert, neurologically intact trauma patients with persistent midline tenderness and negative computed tomography results. Ann Emerg Med.

[15] Chew BG, Swartz C, Quigley MR, Altman DT, Daffner RH, Wilberger JE (2013). Cervical spine clearance in the traumatically injured patient: is multidetector CT scanning sufficient alone?. J Neurosurg Spine.

[16] Steigelman M, Lopez P, Dent D, Myers J, Corneille M, Stewart R, Cohn S (2008). Screening cervical spine MRI after normal cervical spine CT scans in patients in whom cervical spine injury cannot be excluded by physical examination. Am J Surg.

[17] Mavros MN, Kaafarani HM, Mejaddam AY (2015). Additional imaging in alert trauma patients with cervical spine tenderness and a negative computed tomographic scan: is it needed?. World J Surg.

[18] Malhotra A, Durand D, Wu X, Geng B, Abbed K, Nunez DB, Sanelli P (2018). Utility of MRI for cervical spine clearance in blunt trauma patients after a negative CT. Eur Radiol.

[19] Schuster R, Waxman K, Sanchez B, Becerra S, Chung R, Conner S, Jones T (2005). Magnetic resonance imaging is not needed to clear cervical spines in blunt trauma patients with normal computed tomographic results and no motor deficits. Arch Surg.

[20] Novick D, Wallace R, DiGiacomo JC, Kumar A, Lev S, George Angus LD (2018). The cervical spine can be cleared without MRI after blunt trauma: a retrospective review of a single level 1 trauma center experience over 8 years. Am J Surg.

[21] Resnick S, Inaba K, Karamanos E (2014). Clinical relevance of magnetic resonance imaging in cervical spine clearance: a prospective study. JAMA Surg.

[22] Como JJ, Diaz JJ, Dunham CM (2009). Practice management guidelines for identification of cervical spine injuries following trauma: update from the eastern association for the surgery of trauma practice management guidelines committee. J Trauma.

